# Indirect genetic effects contribute substantially to heritable variation in aggression-related traits in group-housed mink (*Neovison vison*)

**DOI:** 10.1186/1297-9686-46-30

**Published:** 2014-05-07

**Authors:** Setegn Worku Alemu, Piter Bijma, Steen Henrik Møller, Luc Janss, Peer Berg

**Affiliations:** 1 Department of Molecular Biology and Genetics, Aarhus University, Tjele DK-8830, Denmark; 2 Animal Breeding and Genomics Centre, Wageningen University,6700AH Wageningen, the Netherlands; 3 Department of Animal Science - Epidemiology and management, Aarhus University, Tjele DK-8830, Denmark; 4 NordGen, Nordic Genetic Resource Center, P.O. Box 115, 1431 Ås, Norway

## Abstract

**Background:**

Since the recommendations on group housing of mink (*Neovison vison*) were adopted by the Council of Europe in 1999, it has become common in mink production in Europe. Group housing is advantageous from a production perspective, but can lead to aggression between animals and thus raises a welfare issue. Bite marks on the animals are an indicator of this aggressive behaviour and thus selection against frequency of bite marks should reduce aggression and improve animal welfare. Bite marks on one individual reflect the aggression of its group members, which means that the number of bite marks carried by one individual depends on the behaviour of other individuals and that it may have a genetic basis. Thus, for a successful breeding strategy it could be crucial to consider both direct (DGE) and indirect (IGE) genetic effects on this trait. However, to date no study has investigated the genetic basis of bite marks in mink.

**Result and discussion:**

A model that included DGE and IGE fitted the data significantly better than a model with DGE only, and IGE contributed a substantial proportion of the heritable variation available for response to selection. In the model with IGE, the total heritable variation expressed as the proportion of phenotypic variance (T^2^) was six times greater than classical heritability (*h*^2^). For instance, for total bite marks, T^2^ was equal to 0.61, while *h*^2^ was equal to 0.10. The genetic correlation between direct and indirect effects ranged from 0.55 for neck bite marks to 0.99 for tail bite marks. This positive correlation suggests that mink have a tendency to fight in a reciprocal way (giving and receiving bites) and thus, a genotype that confers a tendency to bite other individuals can also cause its bearer to receive more bites.

**Conclusion:**

Both direct and indirect genetic effects contribute to variation in number of bite marks in group-housed mink. Thus, a genetic selection design that includes both direct genetic and indirect genetic effects could reduce the frequency of bite marks and probably aggression behaviour in group-housed mink.

## Background

Social interactions among individuals are common both in plants and animals [1] and can have significant effects on production and welfare traits. For example, social interactions can affect feed intake and growth rate in domestic pigs [2,3], lead to mortality due to cannibalism in laying hens [4], result in aggression and tail biting if mixing is carried out in pigs [5], increase competition in fish [6], affect growth rate and disease traits in forestry [7-9], and result in bite marks in mink [10-13]. Because social interactions may have a heritable component, selection acting on these interactions may affect significantly response to artificial selection [14-17]. Therefore, social interactions are a key factor when designing artificial breeding programmes in domestic animals for which group housing is common practise [16].

Results have shown that social interactions among individuals may create additional heritable variation [15]. Ellen et al. [18] found that, in laying hens, total heritable variation in survival days, expressed as the proportion of phenotypic variance, was 1.5 to 3-fold greater than the variance of the direct genetic effect (DGE). Wilson et al. [19] reported that indirect genetic effects (IGE) increased total heritable variation, expressed as a proportion of phenotypic variance, from 0.01 to 0.6 for rearing rate and 0.05 to 0.56 for reciprocal latency rate. These results indicate that more than 80% of the heritable variation of these behavioural traits is due to social interactions [19]. Therefore, for socially affected traits, the heritable variation due to social interactions can be a significant source of heritable variation in domestic, natural, and laboratory populations, for both behavioural traits and production traits [15,16,20,21] and taking such interactions into account may reveal that their genetic variation is significantly greater than previously thought. However, if these interactions are competitive, the heritable variation may be significantly reduced, even to a value of zero when the direct-indirect genetic correlation equals -1 [21,22]. The negative covariance between direct and indirect genetic effect cancels both the direct and indirect genetic effects [21,22].

With the exception of maternal genetic effects, breeders have focused on improving the direct effect of the genotype of the individual on its own phenotype [23]. Hence, the traditional genetic model does not include the social effect of an individual on the phenotypes of its group mates, the so-called Indirect Genetic Effect (IGE; [17,20]). Ignoring IGE may result in a suboptimal response to selection and even a negative response to selection for socially affected traits [17]. For example, individual selection to increase the size of flour beetles populations (*Tribolium castaneum*) decreased the population size in the next generations [24]. Similarly, in non-beak-trimmed laying hens, selection of the survivors decreased survival rate in the next generations [16]. Thus, inclusion of IGE is vital to obtain an optimal response to selection for socially affected traits, which means that the traditional quantitative genetic model should be extended to include the heritable effect of an individual on the phenotypes of its group mates [15-18].

One way of using IGE for response to selection is group selection. It was shown that group selection was effective compared to individual mass selection in decreasing the mortality rate of laying hens, mainly due to aggression, from 68% in generation 2 to 9% in generation 6 [4] and in improving longevity of layers [25]. Another example is the positive response for low leaf and high leaf area in *Arabidopsis thaliana* obtained with group selection versus the negative response with individual selection [26]. The reason for the effectiveness of group selection is that it takes into account part of the IGE.

Although group selection is effective in reducing mortality in chickens and increasing growth in *Arabidopsis thaliana*, it uses only the between-group genetic variance and completely ignores the within-group variance. Thus, group selection is efficient only when group members are sufficiently related [22,27,28]. Moreover, using group selection does not provide any insight into the relative importance of direct *vs.* indirect genetic effects. It is important to understand the genetic parameters that underlie the interactions because it would help to quantify the potential contribution of IGE to response to selection, to estimate breeding values for both direct and indirect genetic effects, and to optimize breeding programmes [14]. This can be achieved by a BLUP (best linear unbiased prediction) model that separates DGE and IGE and gives weights to each of them according to the variance covariance structure of the genetic parameters [2,14-16].

IGE are increasingly important in European mink production because of changes in the housing system from pair-wise to group housing that is becoming more and more frequent. In the wild, juvenile mink leave the mother’s territory at the age of three to four months in order to find their own territory [29,30] and by the end of the growth season, their territorial behaviour is fully developed. This process of dispersal involves increased aggression between the dam and the juveniles as well as between juveniles. The male territory may overlap that of several females but is defended against mink of the same sex [29,31]. Therefore, in Europe during the growth season, juvenile mink are traditionally housed in pairs of one male and one female per cage. In spite of their territorial nature, recommendations on cage sizes for group housing of mink were adopted by the Council of Europe in 1999 [32], probably because welfare improvements were expected from ‘social enrichment’ as discussed in [33]. Group housing has become more and more common because it increases the stocking density in the cages and thereby decreases housing investments. Group housing also increases the social dynamics of the environment which could be a potential disadvantage, since studies on animal welfare in group housing report increased aggression resulting in more bite wounds and bite marks [12,13,34,35].

Direct observation of aggression is time-consuming and it is difficult to distinguish between aggressions and play in mink [36,37]. Thus, it is not a feasible option for collecting the required data for breeding against aggressive behaviours in mink. An alternative solution could be to record the consequences of aggressive behaviours, such as bite marks. Bite marks are the result of a hard pressure to the skin, e.g. a bite, during the 7-week growth phase of the winter coat [38] and, as such, are an excellent indicator of aggression accumulated over this period, and of reduced animal welfare [12,33,39]. In mink, bite marks can occur anywhere on the body and are often scored on the neck, tail and all the body without neck and tail (referred to as “body” in the following), in order to quantify different types of aggressive interactions [11,39]. If inflicting bite marks is a genetically inherited behaviour, then genetic selection that includes both DGE and IGE may be an efficient way to reduce bite marks in group-housed mink. To date, no studies have quantified direct and indirect genetic variation for the number of bite marks in mink.

In this study, we tested the hypothesis that the number of bite marks on different parts of the body is affected by both DGE and IGE. Towards this aim, we estimated the direct and indirect additive genetic (co)variances for the number of bite marks on different regions of the body. Genetic correlations between the numbers of bite marks on different parts of the body were also estimated. Furthermore, we tested whether DGE and IGE on the bite marks in different parts of the body were related to the individual’s body weight, since body weight can be an indicator of social dominance. For instance, a positive genetic correlation between body weight and IGE on bite mark number could indicate that individuals with a dominant genotype for higher weight inflict more bite marks on group mates.

## Methods

### Materials

The consequences of aggressive behaviour in mink (*Neovison vison*) can be recorded by visual observation of injuries i.e. scars on the skin of live animals or dead bodies at pelting, or by the number of bite marks on the flesh side of the skin just after fleshing during the pelting process. The number of bite marks gives an indication of the number of aggressions received by the individual over a period of time prior to pelting.

We used bite marks recorded at pelting as an indirect measure of aggressive behaviour. Bite marks were recorded just after fleshing and after scraping and brushing off sawdust. In 2009, a selection experiment was initiated to select for reduced number of bite marks at pelting, at the mink farm at the Research Centre Foulum in Denmark. We analysed data from the first three generations of that selection experiment. A total of 1985 mink descending from 136 sires and 349 dams were used. Two weeks after weaning i.e. at around 10 weeks post-partum, the juveniles were separated into groups composed of four juvenile mink. Each group of two male siblings and two female siblings was placed in a two storey cage. These procedures were applied in 2009 and repeated in 2010 and 2011. The female siblings were unrelated to the male siblings within a cage except for the 2009 data set, but most individuals had siblings present in other cages. In some cases, data from only three or two mink was obtained mainly because of lack of pedigree information or loss of ID tags during the pelting procedure, and in few cases because of injury or death. Overall, useful data was recorded for two mink from 208 pens, for three mink from 87 pens and all four mink from 327 pens. Individuals were pelted in November 2009, December 2010 and December 2011. At pelting, the number of bite marks on the skin side of the pelt was recorded. The number of bite marks was subjectively measured based on the scale described in Table 1, and expressed as a bite mark score (BMS). From each litter, siblings of the group-housed juveniles were kept in pairs and were the selection candidates. Parents for the next generation were selected from the candidates based on the number of bite marks in their group-housed litter mates. Each individual was selected based on the performance of the mean phenotype of the litter mates’ pen. Thus, the selection method takes into account both DGE and IGE.

**Table 1:** Bite mark score (BMS) used for subjectively measuring the number of bite marks at pelting

**BMS**	**Number of bite marks**
0	0
1	1-5
2	6-10
3	11-15
4	16-20
5	21-25
6	26-30
7	31-35
8	36-45
9	More than 45

**Figure 1:**
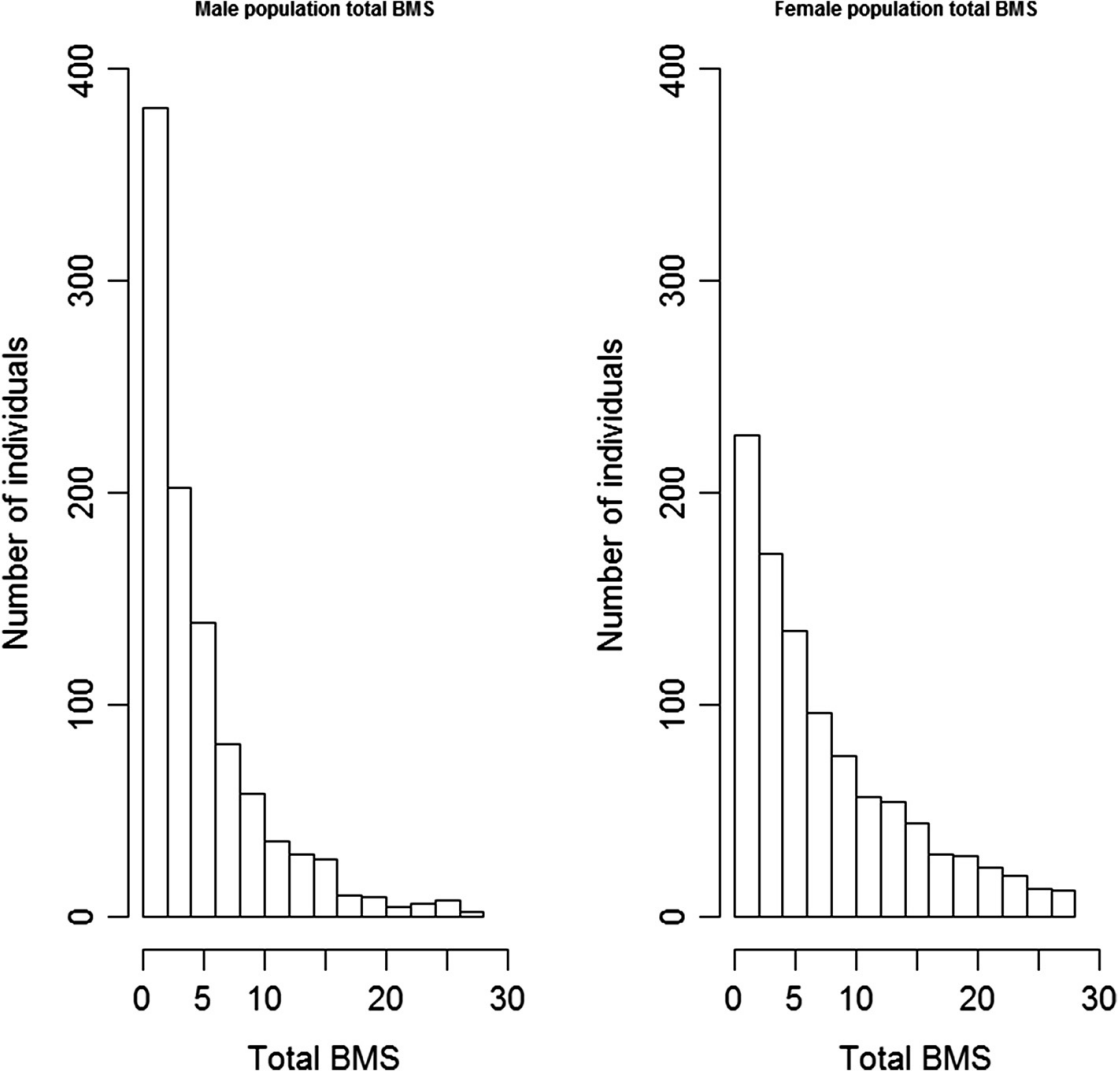
**Histogram of residuals**^**1 **^**of raw data on total BMS**^**2 **^**before transformation**^**3**^**. **^1^Residuals come from a model **y** = **Xb** + **e**, where fixed effects in **Xb** are identical to those used in the mixed model that is explained in the text; ^2^since total BMS is the sum of BMS on the three body regions, it ranges from 0 to 27 (see Table 1); ^3^for the male and female populations, skewness for total BMS corrected for fixed effects was equal to 1.67 and 1.12, respectively and kurtosis was equal to 4.14 and 1.43, respectively.

**Figure 2:**
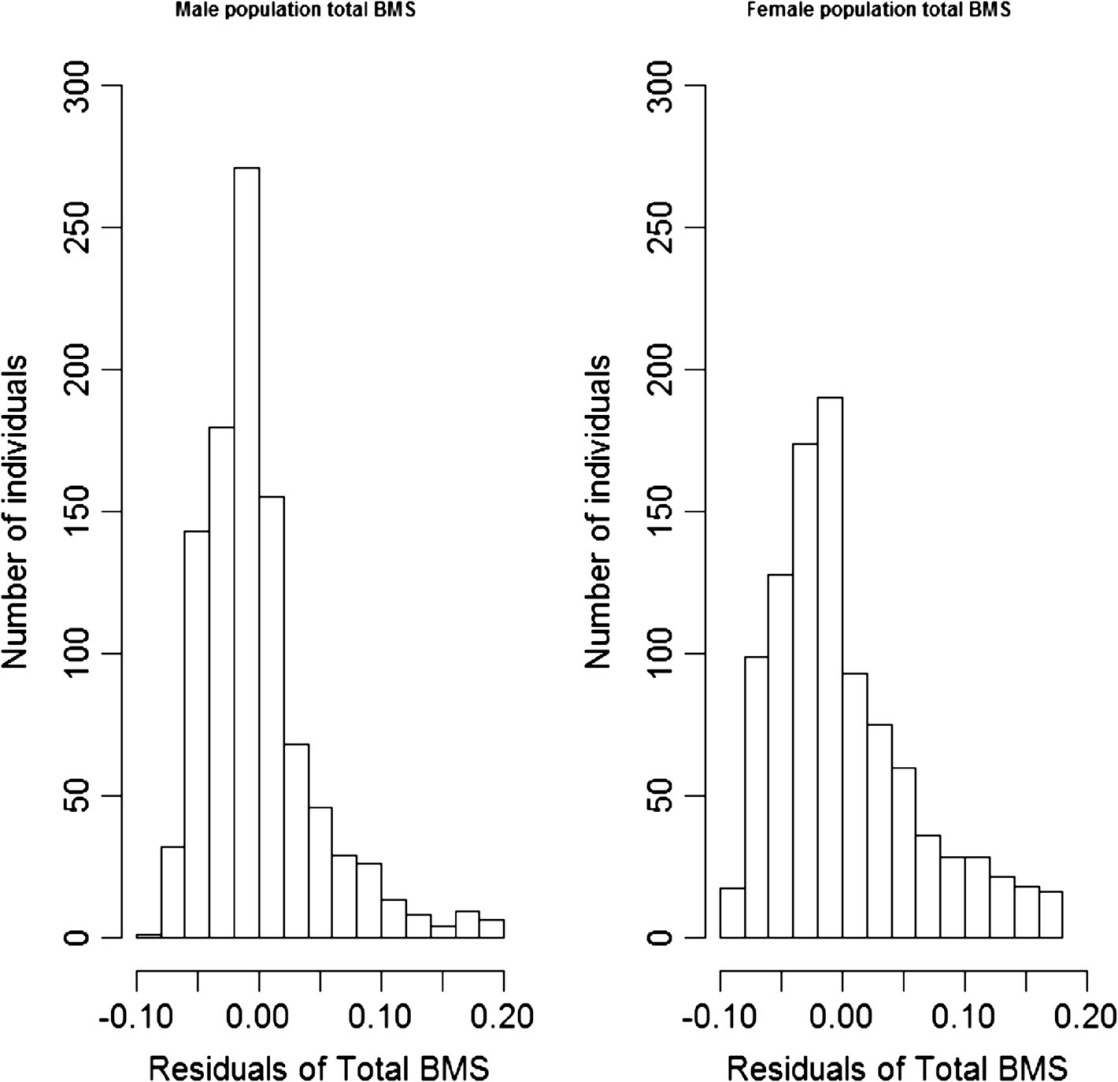
**Histogram of residuals**^**1 **^**for total BMS**^**2 **^**after transformation**^**3**^**. **^1^Residuals come from the model **y** = **Xb** + **e**, where fixed effects in **Xb** are identical to those used in the mixed model explained in the text; ^2^since total BMS is the sum of BMS on the three body regions, it ranges from 0 to 27 (see Table 1), ^3^for the male and female populations, skewness for total BMS corrected for fixed effects was equal to 1.54 and 0.96, respectively and kurtosis was equal to 3.1 and 0.43, respectively; ^2^the transformation was *y*_*t*_ = ln(*y* + 100).

The number of bite marks was scored on the neck (from the nose tip to the shoulder/front leg), body (from the shoulder, including the front legs, to 10 cm above the base of the tail) and tail (from 10 cm above the base of the tail, including the hind legs). A total score was computed as the sum of these three scores. As shown on the histogram in Figure 1, the data were not normally distributed. Log transformation after adding 100 to each observation improved the normality slightly, as illustrated by skewness and kurtosis before and after transformation (Figure 2). Table 2 summarizes BMS per sex for the different parts of the body.

**Table 2:** Mean (standard deviation) of BMS and body weight per sex

**Trait**	**Male**	**Nb individuals**	**Female**	**Nb individuals**
Neck BMS	1.35 (1.62)	996	2.72 (2.33)	986
Body BMS	2.18 (2.53)	992	2.25 (2.34)	984
Tail BMS	1.54 (1.95)	992	2.91 (2.98)	984
Total BMS	5.06 (5.13)	983	7.87 (6.65)	991
Body weight (kg)	2.87 (0.41)	965	1.46 (0.24)	964

Data on BMS and weight were analysed using the GLM procedure in R [40]. This programme was used to decide which fixed effects should be included in the model to estimate the genetic parameters. The following fixed effects i.e. year, sex, number of individuals in a cage (group size; fitted as a factor), and the linear regression on the proportion of male mates per cage (i.e., a covariate, referred to as social sex ratio) were included in the model.

Genetic parameters were estimated using residual maximum likelihood with an animal model [41,42]. Six models were compared with different combinations of random effects. All six models included the DGE of the individual on which the BMS was recorded. The first three models did not include IGE. The first model fitted cage as a random effect, while the second model fitted sex within cage (cage*sex) as a random effect. The reason for fitting cage*sex as a non-genetic random effect, was to test whether social interactions in mink depend on sex. This hypothesis is based on the fact that male mink are usually larger than female mink and thus aggression could occur mainly between cage mates of the same sex. The third model included a cage plus cage*sex random effect. Each of these three models was extended with IGE, giving a total of six models. The best model was selected based on its Akaike information criterion (AIC). In all six models, we used the same fixed effects (see above). Non-genetic maternal effects (common litter effects) were not significant for BMS, and thus were not included in the models. Based on AIC, non-genetic maternal effects were included in the model for body weight. In this section, we present only the most complete model; in the simpler models the relevant terms were omitted. However, we will present results for the two models that had the highest likelihood i.e. one in which IGE were ignored and one in which IGE were included.

The most complete model (referred to as Model 6; see Table 3) was:

$$y=\mathrm{Xb}+Z_{D}a_{D}+Z_{S}a_{S}+\mathrm{Wg}+\mathrm{Vg}*s+e,$$

where **y** is a vector of observed BMS; **b** is a vector of fixed effects, with the incidence matrix **X** linking observations to fixed effects, **Z**_*D*_ and **Z**_*S*_ are known incidence matrices for direct DGE and IGE, and **a**_*D*_ and **a**_*S*_ are vectors of random DGE and IGE, with

$$\left[\begin{array}{c} a_{D}\\ a_{S} \end{array}\right]\sim \mathrm{MVN}\left(0,G⊗A\right),\mathrm{in}\;\mathrm{which}$$

$$G=\left[\begin{array}{cc} \sigma_{A_{D}}^{2} & \sigma_{A_{\mathrm{DS}}}\\ \sigma_{A_{\mathrm{DS}}} & \sigma_{A_{S}}^{2} \end{array}\right],$$

⊗ indicates the Kronecker product, and **A** is the numerator relationship matrix [23,43]. **g** is a vector of random cage effects and **W** the incidence matrix linking records to cages, with $g\sim N\left(0,I_{g}\sigma_{g}^{2}\right)$, where **I**_*g*_ is an identity matrix of appropriate dimension and $\sigma_{g}^{2}$ is the cage variance, **g** * **s** a vector of random cage*sex effects and **V** an incidence matrix, with $g*s\sim N\left(0,I_{g*s}\;\sigma_{g*s}^{2}\right)$, where **I**_*g* * *s*_ is an identity matrix of appropriate dimension and $\sigma_{g*s}^{2}$ is the variance of the cage*sex effect, and **e** is a vector of residuals. We fitted different residual variances for male and female individuals,

$$e=\left[\begin{array}{c} e_{m}\\ e_{f} \end{array}\right]\sim \mathrm{MVN}\left(0,C⊗I_{e}\right),$$

$$C=\left[\begin{array}{cc} \sigma_{e_{m}}^{2} & 0\\ 0 & \sigma_{e_{f}}^{2} \end{array}\right],$$

$$I_{e}=\left[\begin{array}{cc} I_{m} & 0\\ 0 & I_{f} \end{array}\right],$$

where **e**_**m**_ is the vector of residuals for males, and **e**_**f**_ the vector of residuals for females, and $\sigma_{e_{m}}^{2}$ and $\sigma_{e_{f}}^{2}$ are the corresponding variances. In Model 6, the **Z**_*S*_**a**_*S*_ accounts for heritable indirect effects, and **Wg** and **Vg** * **s** account for covariances among cage mates due to non-heritable indirect effects [14,44].

**Table 3:** **Model comparisons using AIC**^**1**^

		**Neck BMS**		**Body BMS**		**Tail BMS**		**Total BMS**	
**Model**	**# Param.**	**Log L**	**AIC**	**Log L**	**AIC**	**Log L**	**AIC**	**Log L**	**AIC**
1. cage	10	-24.9	47.9	-35.3	68.6	-28.0	54.0	-34.1	66.3
2. cage*sex	10	-45.8	89.6	-57.3	112.6	-36.5	70.9	-69.8	137.6
3. cage + cage*sex	11	-24.5	49.0	-33.4	66.8	-16.7	33.4	-31.1	64.0
4. IGE + cage	11	-1.4	2.7	0	0	-3.2	6.4	-0.1	0.2
5. IGE + cage*sex	11	0	0	-0.2	0.4	0	0	0	0
6. IGE + cage + cage*sex	12	0.06	1.9	0.2	1.5	-1	1.3	0.1	1.9

^1^Akaike’s information criterion (AIC) and likelihood value AIC were set to zero as reference for the best model; AIC = 2× # parameters – 2 × log-likelihood; thus lower values indicate a better model.

Genetic parameters for BMS were estimated by implementing the above-mentioned linear animal models in the ASReml software [45]. The matrix of additive genetic relationships **A** was calculated using information on five generations of pedigree that included 2806 animals. Bivariate analysis was also used to estimate the genetic correlation between bite marks on each part of the body, and to estimate the genetic correlation between bite marks and body weight.

### Heritable variation

The above model yields estimates of three genetic parameters, $\sigma_{A_{D}}^{2}$, $\sigma_{A_{\mathrm{DS}}}$ and $\sigma_{A_{S}}^{2}$. Following Bijma [22], these three parameters can be combined into a measure of the total heritable variance that determines the potential of the trait to respond to selection. Since each individual interacts with n-1 group mates, the total heritable impact of an individual’s genes on trait values in the population equals:

(1) $$A_{\;T_{i}}=A_{D_{i}}+\left(n-1\right)A_{S_{i}},$$

where *A* _*T*_ represents the total breeding value, which is a generalization of the traditional breeding value to account for IGE. The total heritable variance is the variance of the total breeding values among individuals,

(2) $$\sigma_{A_{\;T}}^{2}=\sigma_{A_{D}}^{2}+2\left(n-1\right)\sigma_{A_{\mathrm{DS}}}+\left(n-1\right)\sigma_{A_{S}}^{2}.$$

The $\sigma_{A_{\;T}}^{2}$ expresses the heritable variance in absolute units as the additive genetic variance in classical models. The interpretation of $\sigma_{A_{\;T}}^{2}$ becomes easier by expressing heritable variance relative to phenotypic variance, similarly to the classical heritability [46]:

$$T^{2}=\frac{\sigma_{A_{\;T}}^{2}}{\sigma_{P}^{2}}.$$

A comparison of *h*^2^*versus T*^2^ reveals the proportion of the contribution of IGE to the heritable variance that determined the potential of the population to respond to selection.

## Results

Table 4 shows the estimated fixed effects and their statistical significance. The fixed effect year was significant for bite marks on all regions of the body i.e. neck, body, and tail . Sex and social sex ratio were also significant for bite marks on the neck and tail but not on body and group size was significant only for bite marks on the neck.

**Table 4:** Estimated fixed effects and their significance

**Trait**	**Year**	**Sex**^**1**^	**Social sex ratio**^**2**^	**Group size**^**3**^
Neck	***	1.44^**^	-0.20**	(0.63,0.71) *
Body	***	0.01^NS^	0.17^NS^	(1.80, 1.93) ^NS^
Tail	***	1.81^***^	-0.97***	(1.89,1.67) ^NS^
Total	***	1.2***	-0.50**	(1.53,1.54) ^NS^
Body weight	NS	2.7^***^	-0.10^NS^	(-0.33 ,-0.31) *

*, **, *** significant at P ≤ 0.05, 0.01, 0.001, respectively; ^1^the estimate for sex refers to females minus males; ^2^social sex ratio represents the proportion of male group mates of an individual, fitted as a covariable, thus the estimate is the regression coefficient of bite marks on proportion of male group mates in the cage; ^3^the two group size estimates refer to the difference between group size three minus group size two and group size four minus group size two, respectively.

Table 3 (see above) shows the log-likelihood values and AIC for all Models 1 through 6. Based on AIC, the best model among the six tested is Model 5 for bite marks on all regions except body for which Model 4 is slightly better. AIC values show that, in spite of the relatively small dataset, models that included IGE were substantially better than those that did not (Models 1 to 3 *vs*. 4 to 6). Thus, IGE contribute to the heritable variation of BMS on all locations of the body. Models with a random cage*sex effect were the best based on AIC, but differences in AIC between Models 4 to 6 were very small.

Table 5 shows the estimated variance components obtained with the classical model that included direct genetic effects only, but accounted for both cage and cage*sex as non-genetic random effects (Model 3). The genetic parameters were assumed to be the same in both sexes. The additive genetic variance ranged from 0.78 for neck bite marks to 1.15 for tail bite marks. Heritability ranged from 0.18 to 0.23, and differed significantly from zero. We found no common maternal effects for BMS. Non-genetic variances of BMS were higher in females than in males, which agrees with the observation that the mean BMS for females was closer to the middle of the scale used for BMS (Tables 1 and 2).

**Table 5:** **Estimated variance components (±SE) from a traditional animal model ignoring IGE (model 3)**^**1**^

**Parameter**	**Neck BMS**	**Body BMS**	**Tail BMS**	**Total BMS**	**Weight (Kg)**
${\hat{\sigma }}_{A}^{2}$	0.62 ± 0.15	1.06 ± 0.22	0.95 ± 0.19	7.26 ± 1.38	0.06 ± 0.015
${}^{2}\hat{\rho }$	0.28 ± 0.047	0.26 ± 0.04	0.17 ± 0.028	0.26 ± 0.04	-0.15 ± 0.09
${}^{2}{\hat{\rho }}_{s}$	0.05 ± 0.054	-0.09 ± 0.05	-0.17 ± 0.03	-0.09 ± 0.05	0.40 ± 0.19
${\hat{\sigma }}_{e_{m}}^{2}$	1.18 ± 0.12	2.74 ± 0.22	2.31 ± 0.20	11.4 ± 1.14	0.026 ± 0.008
${\hat{\sigma }}_{e_{f}}^{2}$	2.93 ± 0.22	3.53 ± 0.27	5.98 ± 0.34	22.4 ± 1.72	0.03 ± 0.009
${}^{3}{\hat{\sigma }}_{P}^{2}$	3.54 ± 0.11	4.95 ± 0.24	5.31 ± 0.18	31.09 ± 1.00	0.011 ± 0.005
${\hat{h}}^{2}$	0.18 ± 0.04	0.21 ± 0.08	0.18 ± 0.036	0.23 ± 0.04	0.57 ± 0.13
${\hat{c}}^{2}$	-	-	-	-	0.07 ± 0.05

^1^Model 3 was **y** = **Xb** + **Z**_*D*_**a**_*D*_ + **Wg** + **Vg** * **s** + **e**; ^2^although cage and cage*sex covariances were fitted, the result is expressed as the non-genetic correlation between phenotypes of cage mates, $\hat{\rho }=\frac{{\hat{\sigma }}_{g}^{2}}{{\hat{\sigma }}_{g*s}^{2}+{\hat{\sigma }}_{g}^{2}+0.5\;\left({\hat{\sigma }}_{e_{{}_{m}}}^{2}+{\hat{\sigma }}_{e_{f}}^{2}\right)}$*,* and as the non-genetic correlation between phenotypes of cage mates of the same sex, ${\hat{\rho }}_{s}=\frac{{\hat{\sigma }}_{g*s}^{2}}{{\hat{\sigma }}_{g*s}^{2}+{\hat{\sigma }}_{g}^{2}+0.5\;\left({\hat{\sigma }}_{e_{{}_{m}}}^{2}+{\hat{\sigma }}_{e_{f}}^{2}\right)}$ ; ^3^for BMS, phenotypic variance was estimated from a separate analysis using the model **y** = **Xb** + **e**, this was done because our objective was to present a single number for phenotypic variance and heritability, covering both sexes, since a single genetic variance was fitted covering both sexes; however, since our aim was to estimate the other model terms with the best fitting model, a separate analysis for phenotypic variance was performed; the standard errors of heritability estimates were calculated from the full model, averaging the residual variances for both sexes; ${\hat{c}}^{2}=\frac{{\hat{\sigma }}_{n_{d}}^{2}}{{\hat{\sigma }}_{p}^{2}}$, ${\hat{\sigma }}_{n_{d}}^{2}$ refers to the non-genetic dam variance; ${\hat{c}}^{2}$ refers to the non-genetic maternal effect.

The estimated heritability for body weight was equal to 0.58. We found a non-significant ($\sigma_{c}^{2}/\sigma_{P}^{2}=0.07$) common maternal environment effect for body weight. Although the effect is non-significant, it improved the fit of the model for body weight. The cage variance was significantly different from zero for bite marks on all regions of the body. (This conclusion is based on the ratio of the estimate and its SE (standard error), which was much greater than 2). Although the cage*sex-effect was not significantly different from zero for all regions of the body, it was included in the model because it improved the AIC (Table 3). Thus, both cage and cage*sex effects improved the AIC when IGE were ignored.

Table 6 shows the estimated variance components obtained with Model 5 that includes both DGE and IGE and the cage*sex effect. The standard errors on the estimated genetic variances show that both DGE and IGE contributed to variation in BMS. IGE were significantly different from zero for bite marks on all regions of the body and variance of IGE ranged from 0.14 for tail bite marks to 0.27 for body bite marks. The total heritable variation for BMS ranged from 2 to 3.7, and was significantly higher than the additive genetic variance obtained with the traditional model. The total heritable variation expressed as the proportion of phenotypic variance ranged from 0.41 to 0.61, and was ~6 times greater than the direct heritability. The correlation between DGE and IGE of BMS ranged from 0.55 to 0.99 in all parts of the body. Comparison of heritability estimates in Tables 5 and 6 (*h*^2^ and $h_{D}^{2}$) indicates that ordinary heritability estimated with the traditional model overestimates the importance of direct effects by a factor of ~2. In the traditional model, presence of IGE biases the estimate of additive genetic variance upwards. This occurs because cage mates are partly related and thus, an individual receives an IGE from its cage mates that is similar to its own IGE. This in turn increases the covariance between relatives in different cages, which biases heritability upwards [47].

**Table 6:** **Estimated variance components (±SE) for both direct effect and indirect effects using Model 5**^**1**^

**Parameter**	**Neck BMS**	**Body BMS**^**5**^	**Tail BMS**	**Total BMS**
${\hat{\sigma }}_{A_{D}}^{2}$	0.26 ± 0.11	0.37 ± 0.14	0.34 ± 0.13	2.95 ± 0.90
${\hat{\sigma }}_{A_{D,S}}$	0.12 ± 0.04	0.27 ± 0.05	0.21 ± 0.04	1.97 ± 0.30
${\hat{\sigma }}_{A_{S}}^{2}$	0.18 ± 0.04	0.27 ± 0.06	0.14 ± 0.04	1.6 ± 0.32
${}^{2}{\hat{\sigma }}_{A_{T}}^{2}$	1.65 ± 0.25	2.56 ± 0.56	2.19 ± 0.30	19.13 ± 2.40
${\hat{r}}_{A_{\mathrm{DS}}}$	0.55 ± 0.22	0.67 ± 0.21	0.99 ± 0.23	0.90 ± 0.15
${}^{3}{\hat{\rho }}_{s}$	0.09 ± 0.05	-0.04 ± 0.04	-0.09 ± 0.03	-0.02 ± 0.04
${\hat{\sigma }}_{e_{m}}^{2}$	1.40 ± 0.12	3.15 ± 0.21	2.80 ± 0.18	14.8 ± 1.01
${\hat{\sigma }}_{e_{f}}^{2}$	3.07 ± 0.20	3.90 ± 0.25	6.10 ± 0.32	24.77 ± 1.54
${}^{4}{\hat{\sigma }}_{P}^{2}$	3.54 ± 0.11	4.95 ± 0.14	5.31 ± 0.16	31.09 ± 1.00
${}^{5}{\hat{h}}_{D}^{2}$	0.07 ± 0.10	0.07 ± 0.03	0.06 ± 0.02	0.10 ± 0.03
${}^{6}{\hat{T}}^{2}$	0.47 ± 0.08	0.52 ± 0.21	0.41 ± 0.06	0.61 ± 0.08

^1^Model 5 was **y** = **Xb** + **Z**_*D*_**a**_*D*_ + **Z**_*S*_**a**_*S*_ + **Vg** * **s** + **e**; ^2^from Equation 2 using a pen size of 3.18; ${}^{3}\rho_{s}=\frac{{\hat{\sigma }}_{g*s}^{2}}{{\hat{\sigma }}_{g*s}^{2}+0.5\;\left({\hat{\sigma }}_{e_{{}_{m}}}^{2}+{\hat{\sigma }}_{e_{f}}^{2}\right)}$ is the non-genetic correlation between phenotypes of cage mates of the same sex; ^4^ for BMS, phenotypic variance was estimated from a separate analysis using the model **y** = **Xb** + **e**, this was done because our objective was to present a single number for phenotypic variance and heritability, covering both sexes since a single genetic variance was fitted covering both sexes; however, since our aim was to estimate the other model terms with the best fitting model, a separate analysis for phenotypic variance was performed; the standard errors of heritability estimates were calculated from the full model, averaging the residual variances for both sexes; ^5^although Model 4 was slightly better, we presented estimates obtained with Model 5 for reasons of consistency; ${}^{5}{\hat{h}}_{D}^{2}=\sigma_{A_{D}}^{2}/\sigma_{P}^{2}$. ${}^{6}{\hat{T}}^{2}=\sigma_{A_{T}}^{2}/\sigma_{P}^{2}$.

Table 7 shows the genetic correlations between BMS on different regions of the body and body weight. Genetic correlations were positive for all bite mark correlations (direct-direct, direct-indirect, and indirect-indirect). Since the bivariate analysis of total BMS with BMS at specific regions of the body failed to converge, total BMS was removed from Table 7. However, there were small negative genetic correlations between direct effects on BMS and body weight, and between indirect effects on BMS and body weight, some of which were significantly different from zero. Hence, there is a weak indication that heavier individuals are less likely to get involved in aggressive interactions.

**Table 7:** **Genetic correlation estimates (±SE) between bite mark scores**^**1 **^**at different parts of the body and with body weight**

			**Direct**			**Indirect**	
		**Weight**^**2**^	**Neck BMS**	**Body BMS**	**Tail BMS**	**Neck BMS**	**Body BMS**
Direct	Neck BMS	-0.29 ± 0.17					
	Body BMS	-0.08 ± 0.17	0.48 ± 0.22				
	Tail BMS	0.21 ± 0.16	0.57 ± 0.22	0.57 ± 0.22			
Indirect	Neck BMS	-0.05 ± 0.10	0.55 ± 0.22	0.78 ± 0.19	0.89 ± 0.16		
	Body BMS	-0.10 ± 0.10	0.52 ± 0.19	0.67 ± 0.21	0.68 ± 0.22	0.78 ± 0.19	
	Tail^3^ BMS	-0.17 ± 0.10	0.60 ± 0.23	0.85 ± 0.24	0.99 ± 0.23	0.96 ± 0.21	0.99 ± 0.27

^1^The analysis for total BMS did not converge and was thus omitted from this Table; ^2^ there was no evidence for IGE on body weight, thus, weight refers to the direct effect only; genetic correlation of direct total BMS vs. direct weight was equal to -0.28 ± 0.13 and indirect total BMS vs. direct weight was equal to -0.15 ± 0.08; correlation of TBV of total BMS with body weight was - 0.21 ± 0.08.

## Discussion

We have provided evidence that BMS is a heritable trait, and thus can be changed by selective breeding. We found that both DGE and IGE contribute to genetic variation of BMS on all regions of the body. IGE contributed a significant proportion of the heritable variation available for response to selection ($\sigma_{A_{T}}^{2}$). The contribution of IGE variance to total heritable variation, measured by the ratio ${\left(n-1\right)}^{2}\sigma_{A_{S}}^{2}/\sigma_{A_{T}}^{2}$, ranged from 30% for tail bite marks to 52% for neck bite marks, while that of DGE variance was about 16% for all regions of the body. Moreover, there was a strong positive correlation between DGE and IGE, which further increased total heritable variance. Thus, most of the heritable variation in BMS relates to IGE. For instance, for total BMS, the variance in IGE and the direct-indirect genetic covariance together contributed 85% of the heritable variation. Estimated genetic correlations between direct and indirect genetic effects were strong and positive and ranged from 0.55 to 0.99, i.e. significantly different from zero, except for bite marks in the neck region. Thus, these results suggest that if a genotype causes an individual to bite more, it also leads the individual to be more bitten, which, in turn, suggests that an individual benefits from not harming others.

Regarding the non-genetic random effects, the cage*sex effect fitted the data better than the cage effect (except for Body BMS). Ignoring cage*sex effects may cause bias in the estimates of the genetic parameter, which has been reported in previous studies using both simulated [48] and real data [14]. Without fitting cage*sex effects, the estimated variance in both the DGE and the IGE was about 7% lower in our data, indicating a minor effect. This makes sense since the cage*sex effect was not very significantly different from zero.

Both cage and cage*sex effects improved the AIC of the traditional model (Model 3). In contrast, when IGE were included in the model, cage effect did not improve the fit of the model. This suggests that IGE are included in the cage variance when they are not accounted for. We included a cage*sex random effect to allow for stronger interactions between individuals of the same sex within a cage (this was expected based on knowledge of behaviour in mink) [29-31]. Such within-sex interactions might lead to systematic similarities or dissimilarities between cage mates of the same sex. Although we fitted cage and cage*sex as covariances, the results are presented as non-genetic correlations between cage mates and between cage mates of the same sex, for ease of interpretation. The cage*sex correlation was close to zero for all parts of the body (Table 6). This result indicates that the non-genetic direct-indirect correlation is close to zero, since the expected value of *ρ*_*s*_ is calculated as:

$$\rho_{s}=\frac{2\sigma_{E_{\mathrm{DS}}}+\left(n_{\mathrm{sex}}-2\right)\sigma_{E_{S}}^{2}}{\sigma_{E_{D}}^{2}+\left(n-1\right)\sigma_{E_{S}}^{2}}=\frac{2\sigma_{E_{\mathrm{DS}}}}{\sigma_{E_{D}}^{2}+2.18\sigma_{E_{S}}^{2}},$$

where $\sigma_{E_{\mathrm{DS}}}$ is the covariance between direct and indirect non-genetic effects, $\sigma_{E_{D}}^{2}$ is the direct environmental variance, $\sigma_{E_{S}}^{2}$ is the indirect environmental variance, *n* is the number of individuals in a cage, and *n*_sex_ is the number of individuals of the same sex in a cage, which on average was equal to 2 in our data. Thus, in contrast to the clearly positive direct-indirect genetic correlation ($r_{A_{\mathrm{DS}}}$, Table 6), the non-genetic direct-indirect correlation was practically zero.

Given the strong positive direct-indirect genetic correlation, it is surprising that the non-genetic direct-indirect correlation is near zero. However, in our data, group mates of the same sex were full sibs. Thus, the cage*sex correlation not only represents the non-genetic correlation between group mates of the same sex, but also between full sibs and those correlations are fully confounded in our data. The kin selection theory predicts that sibs show less competitive interactions [49], which agrees with observations reported for pigs, where members of the same family fight less compared to unrelated individuals [50,51]. Hence, the apparent difference between the genetic and non-genetic correlations between direct and indirect effects may be due to the fact that information on the non-genetic correlation depends completely on interactions between siblings in our data. The estimated direct-indirect genetic correlation, in contrast, includes interactions among non-kin.

By including the cage*sex correlation, we have, at least partly, accounted for non-genetic-indirect effects that depend on relatedness. However, the indirect genetic effects may also differ between kin and non-kin. Hence, estimated parameters for DGE and IGE may depend on group composition with respect to relatedness. This has proven to be a complex issue that we will explore in a future study.

The direct-direct genetic correlations for BMS on different regions of the body were positive (Table 7), which suggests that an individual that is less bitten on one part of its body is also likely to be less bitten on the other parts of its body. The direct-indirect genetic correlations for BMS on different regions of the body were also positive, which indicates that an individual that is less bitten on one part of its body is less likely to bite other parts of the body of its cage mates. Finally, the indirect-indirect genetic correlations for BMS were also positive, which implies that an individual that bites more or less one part of the body of its cage mates will also bite more or less the other parts of the body of its cage mates. We also investigated the genetic correlations between weight and direct and indirect effects on BMS, but found no significant correlations. Thus, selecting for increased size (larger pelts) animals, which implies increased weight, is not expected to lead to more biting.

Our findings suggest that it is possible to select mink that have a considerably lower level of biting. Irrespective of the selection strategy, response to selection is always equal to the product of the intensity of selection, the accuracy of selection, and the standard deviation of total heritable variation, $R=\mathrm{i\rho}\sigma_{A_{T}}$[22]. For instance, for total BMS, $\sigma_{A_{T}}$ is equal to 4.36 and the mean of total BMS is equal to 6.47, which means that the current total level is only 1.48 genetic standard deviation away from zero. Even with a low accuracy and a moderate intensity, we can produce mink that have a significantly lower level of biting. For instance, with mass selection for total bite marks, which would require recording BMS on live animals, the accuracy is [52]

$$\rho_{T,\mathrm{IS}}=\frac{r\sigma_{A_{T}}^{2}+\left(1-r\right)\left[\sigma_{A_{D}}^{2}+\left(n-1\right)\sigma_{A_{\mathrm{DS}}}\right]}{\sigma_{A_{T}}\sigma_{P}},$$

which equals ~0.4 based on our estimates. Then, if 10% of the population is used for breeding to have an intensity of selection equal to 1.76, the predicted response to selection will be equal to ~3.07 and the total BMS is predicted to reduce from ~6.47 to ~ 3.4, which is a very substantial reduction in a single generation of selection. When using group selection for groups of four sibs, two males and two females that all belong to the same family, it is possible to reach an even higher accuracy i.e. ~ 0.65, and thus the predicted response to selection will be ~5. Using sib selection, which is more appropriate for bite marks since they are recorded on the pelts of dead individuals, the predicted accuracy will be equal to ~ 0.54 and the response to selection to ~4.14. Thus, total BMS will be reduced from ~6.47 to ~ 3.33, again a very substantial reduction in a single generation of selection. In 2011, on average, the difference in total BMS between the selected and control lines was 4.5 in both sexes, which is in reasonable agreement with the range of predicted responses. Thus, although in practice response to selection is usually lower than the theoretical predicted value, our results indicate that it is possible to select mink that have a considerably lower level of biting in a few generations.

## Conclusion

In summary, we confirm the hypothesis that both DGE and IGE contribute to variation in number of bite marks in group-housed mink. Since IGE contribute a substantial amount of heritable variation, genetic selection can reduce bite marks and possibly aggressive behaviour in group-housed minks. Including IGE in selection designs would ensure a more efficient selection against bite marks.

## Competing interests

The authors declare that they have no competing interests.

## Authors’ contributions

PBe initiated the research. SWA analysed the data, wrote the draft and final paper. PBi provided guidance to analyse the data, interpret the output, and helped to write the paper. PBe and SHM collected the data and helped to write the paper. LJ helped to write the paper. All authors read and approved the final manuscript.
