# Erratum to: indirect genetic effects contribute substantially to heritable variation in aggression-related traits in group-housed mink (*Neovison vison*)

**DOI:** 10.1186/s12711-014-0070-8

**Published:** 2014-10-15

**Authors:** Setegn Worku Alemu, Piter Bijma, Steen Henrik Møller, Luc Janss, Peer Berg

**Affiliations:** Department of Molecular Biology and Genetics, Aarhus University, Tjele, DK-8830 Denmark; Animal Breeding and Genomics Centre, Wageningen University, 6700AH Wageningen, the Netherlands; Department of Animal Science - Epidemiology and management, Aarhus University, Tjele, DK-8830 Denmark; NordGen, Nordic Genetic Resource Center, P.O.Box 115, 1431 Ås, Norway

## Erratum

After publication of this work [1], we noted that on page 5, in the second column, the three equations:

$$ e=\left[\begin{array}{c}\hfill {\mathbf{e}}_m\hfill \\ {}\hfill {\mathbf{e}}_f\hfill \end{array}\right]\sim MVN\left(\mathbf{0},\mathbf{C}\otimes {\mathbf{I}}_{\mathbf{e}}\right), $$

$$ \mathbf{C}=\left[\begin{array}{cc}\hfill {\sigma}_{e_m}^2\hfill & \hfill 0\hfill \\ {}\hfill 0\hfill & \hfill {\sigma}_{e_f}^2\hfill \end{array}\right], $$

$$ {\mathrm{I}}_{\mathrm{e}}=\left[\begin{array}{cc}\hfill {\mathbf{I}}_m\hfill & \hfill 0\hfill \\ {}\hfill 0\hfill & \hfill {\mathbf{I}}_f\hfill \end{array}\right], $$

should be removed, and replaced by the following two equations:

$$ e=\left[\begin{array}{c}\hfill {\mathbf{e}}_m\hfill \\ {}\hfill {\mathbf{e}}_f\hfill \end{array}\right]\sim MVN\left(\mathbf{0},\mathbf{E}\right), $$

$$ \operatorname{var}\left(\mathbf{E}\right)=\left[\begin{array}{cc}\hfill {\mathbf{I}}_m{\sigma}_m^2\hfill & \hfill 0\hfill \\ {}\hfill 0\hfill & \hfill {\mathbf{I}}_f{\sigma}_f^2\hfill \end{array}\right]. $$
